# Pseudorabies virus infection triggers pUL46-mediated phosphorylation of connexin-43 and closure of gap junctions to promote intercellular virus spread

**DOI:** 10.1371/journal.ppat.1012895

**Published:** 2025-01-21

**Authors:** Alexander Tishchenko, Nicolás Romero, Cliff Van Waesberghe, Jonas L. Delva, Oliver Vickman, Gregory A. Smith, Thomas C. Mettenleiter, Walter Fuchs, Barbara G. Klupp, Herman W. Favoreel

**Affiliations:** 1 Department of Translational Physiology, Faculty of Veterinary Medicine, Ghent University, Merelbeke, Belgium; 2 Department of Microbiology, Blavatnik Institute, Harvard Medical School, Boston, Massachusetts, United States of America; 3 Department of Microbiology-Immunology, Feinberg School of Medicine, Northwestern University, Chicago, Illinois, United States of America; 4 Institute of Molecular Virology and Cell Biology, Friedrich-Loeffler-Institute, Insel Riems, Germany; State University of New York Upstate Medical University, UNITED STATES OF AMERICA

## Abstract

Gap junctions (GJs) play a pivotal role in intercellular communication between eukaryotic cells, including transfer of biomolecules that contribute to the innate and adaptive immune response. However, if, how and why viruses affect gap junction intercellular communication (GJIC) remains largely unexplored. Here, we describe how the alphaherpesvirus pseudorabies virus (PRV) triggers ERK1/2-mediated phosphorylation of the main gap junction component connexin 43 (Cx43) and closure of GJIC, which depends on the viral protein pUL46. Consequently, a UL46null PRV mutant is unable to phosphorylate Cx43 or inhibit GJIC and displays reduced intercellular spread, which is effectively rescued by pharmacological inhibition of GJIC. Intercellular spread of UL46null PRV is also rescued by inhibition of the stimulator of interferon genes (STING), suggesting that pUL46-mediated suppression of GJIC contributes to intercellular virus spread by hindering intercellular communication that activates STING. The current study identifies key viral and cellular proteins involved in alphaherpesvirus-mediated suppression of GJIC and reveals that GJIC inhibition enhances virus intercellular spread, thereby opening new avenues for the design of targeted antiviral therapies.

## Author summary

Gap junctional intercellular communication (GJIC) is a key feature of multicellular organisms, that allows individual cells to coordinate their functions. Connexin-43 (Cx43) is the most abundant gap junction component. Viruses often exploit cellular processes to enhance their survival and spread, but the role of gap junctions during viral infections and the impact of virus infection on GJIC are incompletely understood. We discovered that pseudorabies virus (PRV), a porcine alphaherpesvirus related to human herpes simplex virus, triggers phosphorylation of Cx43 via the viral pUL46 protein and host ERK1/2 kinase. This modification of Cx43 suppresses GJIC between infected and neighbouring cells, allowing the virus to evade innate immune defences and spread more effectively. A genetically modified PRV that lacks pUL46 displays reduced spread to neighbouring cells, which can be rescued by drugs that either block GJIC or a specific innate antiviral immune pathway termed STING. Our findings reveal a novel mechanism by which viruses manipulate cellular communication to promote virus spread. These insights may guide the development of new antiviral therapies and improve the design of vaccines or oncolytic viruses—engineered viruses used to treat cancer.

## Introduction

Direct cell-to-cell communication is a crucial function of multicellular organisms and is critically involved in, e.g., developmental processes, tissue homeostasis and responses to infectious agents. Gap junction-based communication is one of the most important and best characterised modes of intercellular communication. Gap junctions (GJs) bridge opposing plasma membranes of neighbouring cells and are established by interconnecting oligomers of connexin (Cx) proteins, of which Cx43 is the most widely expressed and best characterized member [1,2]. Alignment of Cx43 sequences from different vertebrate species reveals a high degree of sequence conservation, which indicates the functional importance of this protein [3].

Gap junction intercellular communication (GJIC) is regulated by a well-orchestrated array of post-translational modifications (PTMs) of connexins, which exert their influence either directly, impacting channel gating, or indirectly, by regulating processes like channel folding, trafficking, assembly, docking, and degradation [4]. Phosphorylation of Cx43 is by far the best studied Cx PTM and has been often associated with GJ closure, internalization, and degradation [5–8].

Besides metabolically synchronizing neighbouring cells, GJs play a central role in both adaptive and intrinsic immune responses, particularly against viruses. With regard to the innate antiviral response, Ablasser and colleagues discovered that GJs provide bystander immunity by transferring antiviral second messenger molecules, like 2’3’-cyclic GMP-AMP (cGAMP), from virus-infected to GJ-coupled neighbouring cells [9]. In these neighbouring cells, cGAMP triggers the activation of the endoplasmic reticulum (ER)-resident stimulator of interferon genes (STING) [10], which induces a potent antiviral state in these neighbouring cells [11]. Regarding the adaptive antiviral immune response, Neijssen *et al.* showed that GJs allow intercellular passage of viral peptide antigens that can then be cross-presented to CD8+ cytotoxic T lymphocytes by the recipient cells [12].

Viruses, in particular large DNA viruses like herpesviruses, are notoriously known to hijack and co-opt key cellular processes in infected cells to suppress the immune response and facilitate virus replication and spread [13]. Despite the contribution of GJIC to the antiviral response, to date, only a handful of studies have described the effects of herpesvirus infections on the host’s GJIC. Infection of glioblastoma cells with the betaherpesvirus human cytomegalovirus (HCMV) results in proteasomal Cx43 degradation and reduced GJIC. This process depends on the viral IE72 and IE86 proteins, but the underlying mechanism and potential consequences remain unclear [14]. The IE1 protein of HCMV has also been described to trigger proteasomal Cx43 degradation in HCMV-infected neural progenitor cells (NPC) although its effect on GJIC has not been assessed [15]. There are also indications that infection of epithelial cells with the alphaherpesvirus herpes simplex virus type 2 (HSV-2) results in decreased GJIC, although no responsible viral or host proteins have been identified and the impact on virus replication or spread is not known [16,17]. Hence, although there are indications that herpesviruses may affect GJIC, in general, the underlying mechanisms and functional consequences are poorly understood.

In the current study, we report that infection of epithelial cells with the porcine alphaherpesvirus pseudorabies virus (PRV) leads to pUL46-dependent and ERK1/2-mediated phosphorylation of Cx43, which is associated with suppression of GJIC. We found that PRV pUL46-induced GJIC suppression contributes to efficient intercellular virus spread, and that the impaired intercellular spread of UL46null PRV can be rescued by inhibition of STING. The current data identify key viral and host proteins involved in PRV-mediated suppression of GJIC and highlight interference with GJIC as a novel viral mechanism to enhance intercellular spread.

## Results

### PRV infection results in downregulation of GJIC

To assess whether PRV infection triggers changes in GJIC, a fluorescence-based scrape-loading dye-transfer (SL-DT) assay was performed using a commonly used cell line for this purpose, the WB-F344 rat hepatocyte cell line [18,19]. As a control to confirm that the SL-DT assay works properly, we assessed GJIC in mock-infected WB-F344 cells in the absence or presence of the GJIC inhibitor meclofenamate sodium [20] (S1 Fig).

**Fig 1:**
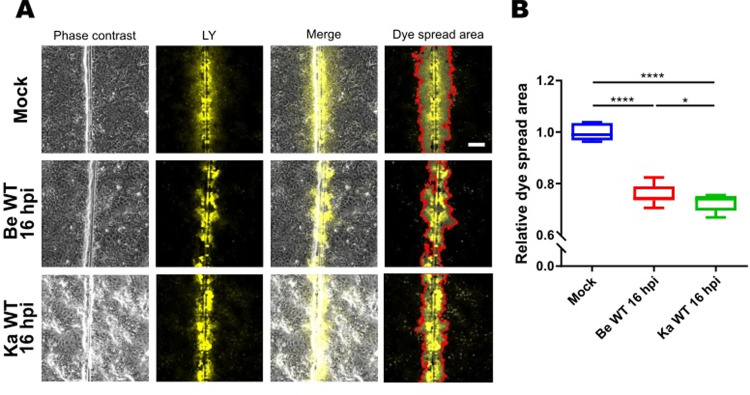
PRV infection results in suppression of gap junctional intercellular communication. (A) Representative images of scrape loading dye transfer (SL-DT) assay performed in WB-F344 cells that were mock-infected or infected for 16h with WT PRV strains Becker or Kaplan (MOI, 10 PFU/cell). Scale bar: 100 μm. (B) Quantitative analysis of SL-DT assay shown in Fig 1A. Dye spread area was normalized to dye spread in mock-infected cells (set to 1). Graphs represent mean and standard deviations of three independent repeats (‘ns’ not significant, ‘****’ P ≤ 0.0001).

As shown in Fig 1, infection of WB-F344 cells for 16h with either of two widely used wild type (WT) PRV strains, Becker and Kaplan, significantly downregulates GJIC (Fig 1A and 1B). Given that both strains suppress GJIC in infected cells, this indicates cross-strain conservation of this phenotype.

### PRV infection of epithelial cells triggers phosphorylation of Cx43, followed by Cx43 degradation

**Fig 2:**
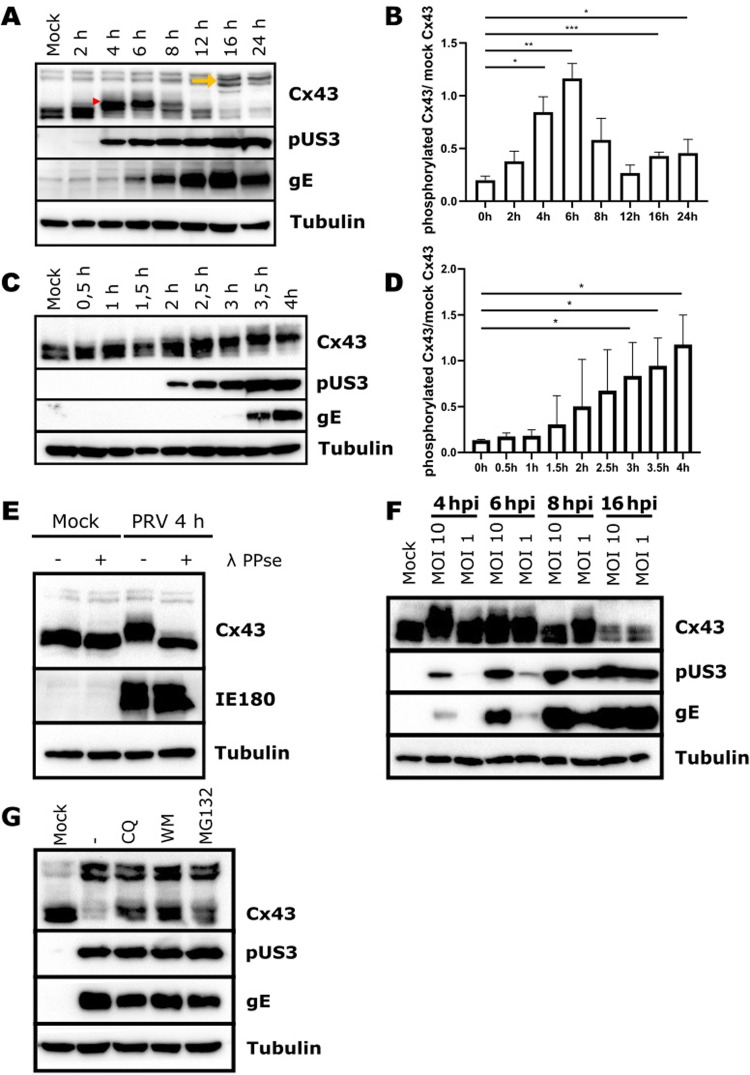
PRV infection results in phosphorylation and subsequent degradation of gap junction protein connexin 43 (Cx43). (A) Western blot analysis of Cx43 SDS-PAGE mobility during a time-course assay performed in ST cells infected with WT PRV strain NIA3 (MOI, 10 PFU/cell) from 0 to 24hpi. (B) Quantification of Cx43 phosphorylation (= upshifted Cx43 indicated with a red arrowhead in (A)) during the time-course shown in Fig 2A. (C) Western blot analysis of Cx43 SDS-PAGE mobility during a time-course assay performed in ST cells infected with WT PRV strain NIA3 (MOI, 10 PFU/cell) from 0 to 4hpi. (D) Quantification of Cx43 phosphorylation during the time-course shown in Fig 2C. (E) Western blot analysis of Cx43 SDS-PAGE mobility in mock-infected or PRV WT strain NIA3-infected ST cells (MOI, 10 PFU/cell, 4hpi) incubated or not with bacteriophage lambda protein phosphatase (λ PPse). (F) Western blot analysis of Cx43 phosphorylation at 4, 6, 8, and 16hpi in ST cells infected with WT PRV strain Becker using an MOI 10 or 1. (G) Western blot analysis of PRV-induced Cx43 degradation in the absence or presence of different inhibitors. ST cells were infected with WT PRV strain NIA3 (MOI, 10 PFU/cell). At 8hpi, the following inhibitors were added: - = no inhibitor, CQ = cloroquine 100 μM, WM = wortmannin 750 nM, MG-123 10 μM. Cell lysates were harvested at 18hpi. All Western blots shown in this figure are representative examples from three independent repeats of each experiment. Red arrowhead denotes upshifted phosphorylated Cx43. Yellow arrowhead denotes slowly migrating high MW Cx43.

To mechanistically understand how PRV infection suppresses GJIC, we assessed by Western blot whether PRV infection affects the main connexin isoform Cx43. A time-course assay showed that PRV infection of porcine epithelial swine testicle (ST) cells affects electrophoretic mobility of Cx43 (Fig 2A and 2B, red arrowhead in Fig 2A denotes the upshifted Cx43 band). A more fine-grained time-course assay early in infection showed that the initial upshift in apparent Cx43 molecular weight can be observed as soon as 2hpi (Fig 2C and 2D), reaching a maximum around 4hpi to 6hpi (Fig 2A–2D). Following this upshift in electrophoretic mobility, Cx43 protein levels are gradually downregulated, resulting in an almost complete lack of detectable Cx43 at 16hpi (Fig 2A and 2B). In addition, we observed an increase in slow migrating Cx43 bands late in infection (marked with a yellow arrow in Fig 2A), which are very similar to earlier described ubiquitinated forms of Cx43 [21]. Inhibition of the ubiquitin-activating enzyme E1 (UAC1) by addition of PYR41 reduced the intensity of the slow migrating Cx43 bands, suggesting that these bands may be caused by ubiquitination (S2A Fig).

Phosphorylation is the most common PTM of connexins [4] and results in a similar upshift in apparent molecular weight of the protein as observed during PRV infection [22,23]. To assess whether the PRV-induced shift in Cx43 electrophoretic mobility reflects Cx43 phosphorylation, we treated lysates of mock- or PRV-infected cells collected at 4hpi with λ protein phosphatase (λ-PPse). Fig 2E shows that dephosphorylation reversed the PRV-induced upshift in Cx43 electrophoretic mobility, confirming that PRV infection triggers phosphorylation of Cx43.

Cx43 phosphorylation was observed using either of three widely used PRV strains (Becker, Kaplan, and NIA-3) (S2B Fig) and in additional porcine epithelial cell types, including the SK-6 epithelial cell line and primary porcine kidney (PPK) epithelial cells (S2C and S2D Fig), indicating that this phenomenon does not depend on virus strain or porcine epithelial cell type. Infection of ST cells with WT PRV using two different multiplicities of infection (MOI of 10 and MOI 1) showed that Cx43 phosphorylation can also be observed when using a lower infectious dose, albeit with somewhat delayed kinetics (Fig 2F).

PRV-induced Cx43 phosphorylation is followed by Cx43 downregulation later in infection (Fig 2A). Phosphorylation has been described before to trigger GJ internalization and degradation [5,6,24] via autophagosomal [25], endolysosomal [25,26], as well as proteasomal [27] pathways. Inhibitors targeting these different pathways (chloroquine diphosphate, wortmannin, and MG132 to inhibit autophagy, lysosomal degradation and the 26S proteasome, respectively) all noticeably but incompletely impaired PRV-induced Cx43 degradation (Fig 2G), suggesting that PRV-induced Cx43 degradation late in infection may occur via different degradation pathways.

### PRV infection triggers Cx43 phosphorylation via activation of the mitogen-activated protein kinases ERK1/2

**Fig 3:**
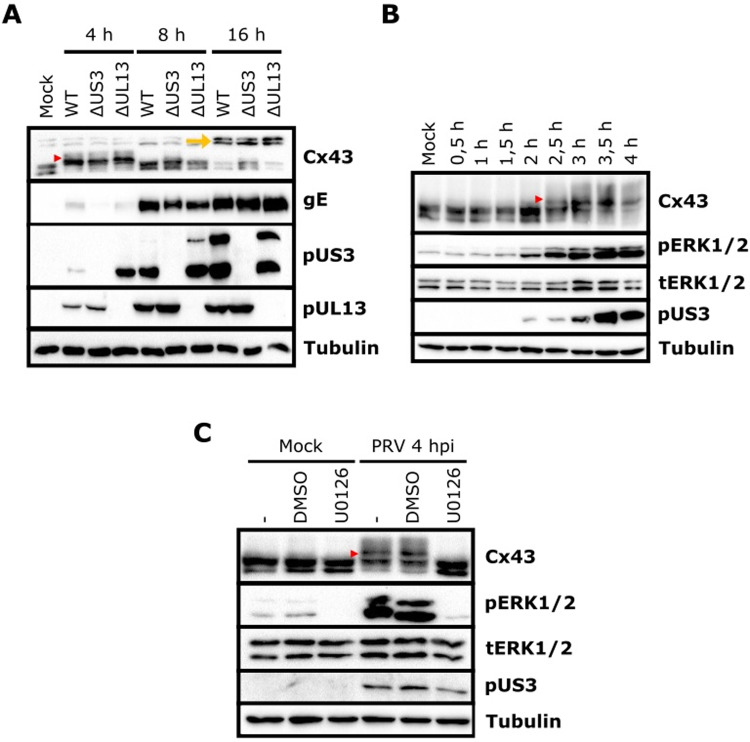
PRV-induced Cx43 phosphorylation is not caused by the viral protein kinases pUS3 or pUL13 but depends on the cellular kinase ERK1/2. (A) Western blot analysis of Cx43 phosphorylation at 4, 8, and 16hpi in ST cells infected with WT PRV strain NIA-3, an isogenic ΔUS3 mutant, or an isogenic ΔUL13 mutant (MOI, 10 PFU/cell). (B) Western blot analysis of total ERK1/2 (tERK1/2) kinase and active ERK1/2 kinase (pERK1/2, phosphorylated at Thr202/Tyr204) during a time-course assay in ST cells infected with WT PRV strain Becker (MOI, 10 PFU/cell) from 0 to 4hpi. (C) Western blot analysis of Cx43 phosphorylation in mock- and WT PRV strain Becker (MOI, 10 PFU/cell)-infected ST cells, treated or not with 50 μM MEK1/2 inhibitor U0126 (added at 1.5hpi). DMSO was used as vehicle control (added at 1.5hpi). All Western blots shown in this figure are representative examples from three independent repeats of each experiment. Red arrowheads denote upshifted phosphorylated Cx43. Yellow arrowhead denotes slowly migrating high MW Cx43.

Alphaherpesviruses encode two highly conserved and multifunctional serine/threonine kinases, pUS3 and pUL13, that have been reported to phosphorylate different viral and host proteins [28]. To assess whether either of the viral kinases is involved in PRV-induced Cx43 phosphorylation, we performed time-course infection assays using WT PRV as well as isogenic US3null and UL13null strains. Fig 3A shows that PRV-induced Cx43 phosphorylation does not rely on its viral kinases, suggesting that cellular kinases are involved in PRV-induced Cx43 phosphorylation.

The mitogen-activated protein kinases ERK1 and ERK2 (ERK1/2) have been extensively characterized to phosphorylate Cx43, and ERK1/2-mediated phosphorylation of Cx43 is strongly associated with GJ closure, internalization, and degradation [29-31]. Interestingly, PRV infection has been reported before to trigger ERK1/2 activation [32-34]. A time-course assay showed that ERK1/2 is phosphorylated early in infection, starting at approximately 2hpi, coinciding with the start of Cx43 phosphorylation (Fig 3B). Treatment of cells with U0126, an inhibitor of MEK1/2 (the kinase that activates ERK1/2) restored the electrophoretic mobility of Cx43 in PRV-infected cells to that in mock-infected cells (Fig 3C). Hence, PRV-induced Cx43 phosphorylation depends on activation of ERK1/2.

### The pUL46 tegument protein of PRV is required for ERK1/2 activation and Cx43 phosphorylation in infected cells and UL46null PRV is unable to suppress GJIC

**Fig 4:**
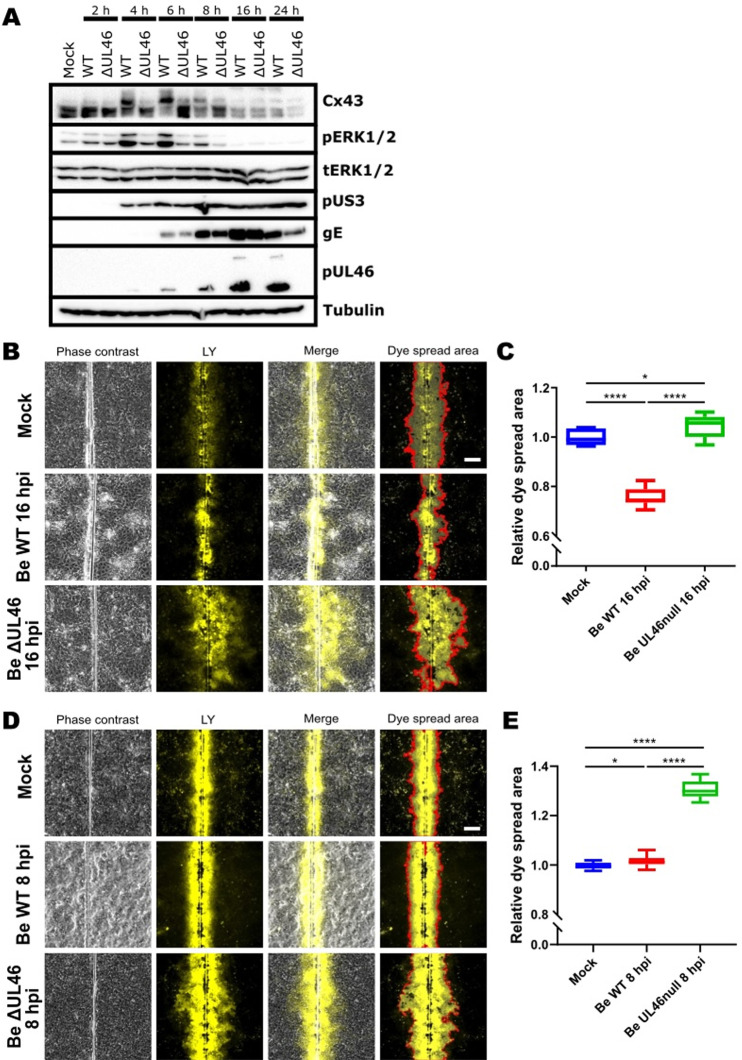
The pUL46 protein is required for PRV-induced phosphorylation of Cx43 and suppression of GJIC. (A) Western blot analysis of Cx43 phosphorylation and ERK1/2 activation during a time-course assay in ST cells infected with WT PRV strain Becker or an isogenic UL46null mutant (MOI, 10 PFU/cell) from 0 to 24hpi. (B) SL-DT assay performed in WB-F344 cells that were mock-infected or infected for 16h with WT PRV strain Becker or with an isogenic UL46null mutant (MOI, 10 PFU/cell). Scale bar: 100 μm. (C) Quantitative analysis of SL-DT assay shown in Fig 4B. (D) SL-DT assay performed in WB-F344 cells that were mock-infected or infected for 8h with WT PRV strain Becker or with an isogenic UL46null mutant (MOI, 10 PFU/cell). Scale bar: 100 μm. (E) Quantitative analysis of SL-DT assay shown in Fig 4D. For the quantification of SL-DT assays dye spread area was normalized to dye spread in mock-infected cells (set to 1). Graphs represent mean and standard deviations of three independent repeats (‘*’ P *<* 0.05, ‘****’ P ≤ 0.0001). All Western blot and fluorescence images shown in this figure were taken from one representative assay out of three independent repeats of each experiment.

We reported earlier that the PRV pUL46 tegument protein triggers ERK1/2 phosphorylation, although the functional consequences remained unclear [34]. To assess whether pUL46 contributes to Cx43 phosphorylation, time-course infection assays were performed on ST cells using WT PRV Becker and an isogenic UL46null PRV mutant. As a control, titration assays at 16 and 24hpi showed that viral replication efficiency was comparable for WT and UL46null PRV (S3 Fig). Fig 4A shows that infection with UL46null PRV resulted in a virtual absence of ERK1/2 activation and a lack of Cx43 phosphorylation. We confirmed these findings using another, independently generated UL46null mutant made in the Kaplan genetic background, which yielded similar results (S4A Fig). These findings reveal that pUL46 is essential for PRV-induced ERK1/2 activation and Cx43 phosphorylation.

We confirmed that PRV-induced Cx43 phosphorylation depends on pUL46 in PRV-infected WB-F344 cells (S4B Fig). In WB-F344 cells, the shift in electrophoretic mobility associated with Cx43 phosphorylation was less clear compared to that in ST cells. Therefore, pUL46-induced phosphorylation of Cx43 in PRV-infected WB-F344 cells was confirmed further via Phos-tag gel analysis (which increases phosphorylation-dependent differences in electrophoretic mobility) (S4B Fig). Phos-tag gels confirmed that PRV also triggers pUL46-dependent Cx43 phosphorylation in WB-F344 cells, which was particularly evident at 8hpi and 12hpi. Infection with UL46null PRV in WB-F344 cells results in less Cx43 degradation late in infection compared to WT PRV-infected cells (S4B Fig), suggesting that phosphorylation may contribute to targeting Cx43 for degradation, at least in some cell types, which is in line with other studies [29-31].

To assess whether pUL46 contributes to PRV-induced suppression of GJIC, SL-DT assays were performed on WB-F344 cells infected with WT PRV strain Becker or its isogenic UL46null mutant. SL-DT assays performed at 16hpi showed that, unlike WT PRV, UL46null PRV infection did not suppress GJIC (Fig 4B and 4C). In addition, GJIC in UL46null PRV-infected cells was slightly but significantly increased compared to that observed in mock-infected cells (Fig 4B and 4C).

To assess whether Cx43 phosphorylation, rather than subsequent Cx43 degradation, contributes to PRV pUL46-mediated inhibition of GJIC, we performed additional SL-DT assays in WB-F344 cells at 8hpi, a time point when pUL46-induced Cx43 phosphorylation is evident without signs of Cx43 degradation (S4B Fig). These assays at 8hpi confirmed our findings at 16hpi. Also, at 8hpi, UL46null PRV-infected cells displayed a substantially higher GJIC compared to WT PRV-infected and to mock-infected cells (Fig 4D and 4E). The increase in GJIC in UL46null PRV-infected cells compared to mock-infected cells was substantially more pronounced at 8hpi compared to 16hpi.

The results of these SL-DT assays at both 8hpi and 16hpi were again confirmed using the independently generated Kaplan strain-based UL46null mutant (S4C and S4D Fig). In the Kaplan genetic background, the increase in GJIC in UL46null PRV-infected cells compared to that in mock-infected cells was observed only at 8hpi (S4C and S4D Fig).

As a conclusion, lack of pUL46 leads to increased GJIC in PRV-infected cells, indicating that pUL46 is required for PRV-induced suppression of GJIC. In addition, particularly at 8hpi, lack of pUL46 results in increased GJIC in PRV-infected cells compared to that in mock-infected cells, indicating that cells trigger increased GJIC in response to PRV infection in the absence of pUL46.

### UL46null PRV displays reduced intercellular virus spread, which can be rescued by GJIC inhibition or by STING inhibition

**Fig 5:**
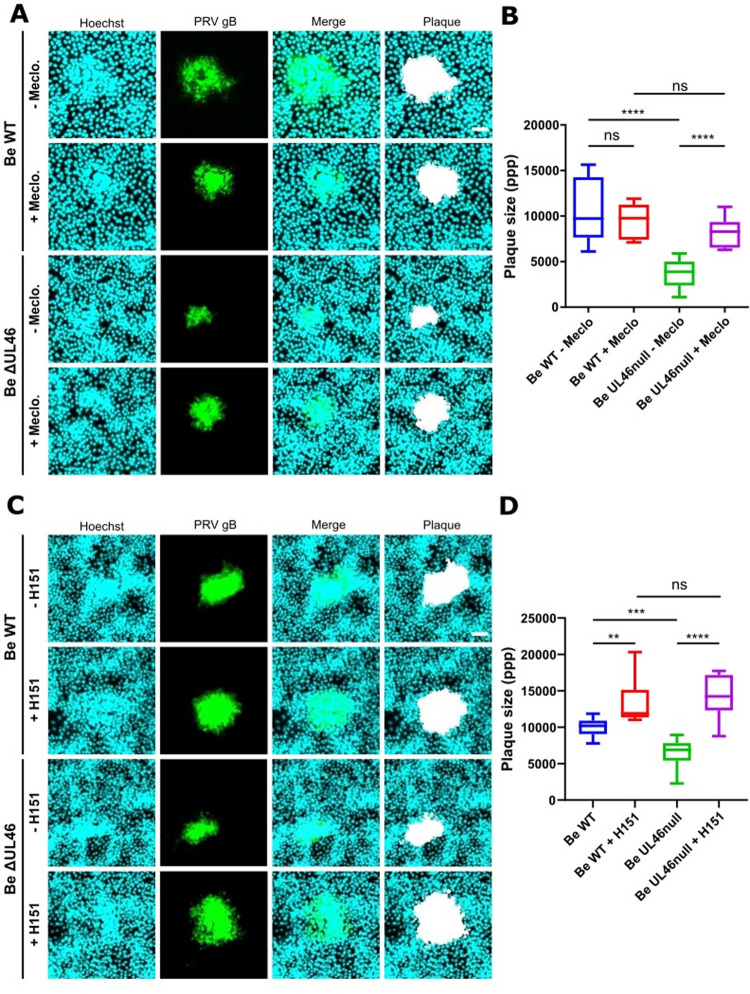
UL46null PRV displays impaired cell-to-cell spread, which can be rescued by GJIC inhibition as well as by STING inhibition. (A) Plaque assay performed in WB-F344 cells infected with PRV WT strain Becker or an isogenic UL46null mutant. 5,000 plaque forming units (PFUs) were used per well of a 6-well-plate to infect WB-F344 cells. At 2hpi, media containing 2% carboxymethyl cellulose and supplemented or not with 50 μM of the GJIC inhibitor meclofenamate sodium was added. Cells were fixed at 24hpi, and stained against PRV gB. Scale bar: 50 μm. (B) Quantitative analysis of plaque sizes shown in Fig 5A (Y-axis represents number of pixels per plaque (ppp)). (C) Plaque assay performed in WB-F344 cells infected with PRV WT strain Becker or an isogenic UL46null mutant. 5,000 plaque forming units (PFUs) were used per well of a 6-well-plate to infect WB-F344 cells. At 2hpi, media containing 2% carboxymethyl cellulose and supplemented or not with 15 μM of the STING inhibitor H-151 was added. Cells were fixed at 24hpi, and stained against PRV gB. Scale bar: 50 μm. (D) Quantitative analysis of plaque sizes shown in Fig 5C (Y-axis represents number of pixels per plaque (ppp)). Graphs represent mean and standard deviations of three independent repeats (‘ns’ not significant, ‘**’ P ≤ 0.01, ‘***’ P ≤ 0.001 ‘****’ P ≤ 0.0001). Immunofluorescence images shown in this figure were taken from one representative assay out of three independent repeats of each experiment. At least 10 plaques per condition were measured during each experimental repeat.

To assess whether the ability of PRV infection to suppress GJIC contributes to intercellular virus spread, virus plaque assays were performed in WB-F344 cells using WT PRV or isogenic UL46null PRV (both in the Becker and Kaplan genetic backgrounds), in the presence or absence of the gap junction inhibitor meclofenamate sodium [35-38]. UL46null PRV shows a reduced ability to form plaques compared to WT PRV, indicating that UL46null is impaired in its ability to spread from infected cells to neighbouring cells (Fig 5A and 5B, and S5A and S5B Fig), which is in line with earlier reports [39,40]. Importantly, both in the Becker and Kaplan genetic background, addition of the GJIC inhibitor meclofenamate sodium increased plaque size in UL46null PRV-infected cells to the level of that observed using WT PRV, while no increase in plaque size was observed when WT PRV-infected cells were treated with meclofenamate sodium (Fig 5A and 5B, and S5A and S5B Fig). Hence, these data show that pUL46-induced suppression of GJIC significantly contributes to the ability of the virus to spread from cell-to-cell *in vitro*, thereby revealing a novel aspect of virus intercellular spread.

Lastly, considering the role of GJs in the spread of antiviral secondary messengers such as cGAMP that activate STING [9,41], we sought to investigate whether STING inhibition could rescue the defect in cell-to-cell spread of UL46null PRV. To this end, plaque assays were performed in WB-F344 cells using WT PRV or isogenic UL46null PRV, again both in the Becker and Kaplan genetic backgrounds, in the presence or absence of the most commonly used STING inhibitor, H-151 [42,43]. Addition of H-151 significantly increased plaque size both for WT and UL46null PRV (Fig 5C and 5D, and S5C and S5D Fig). However, in case of UL46null, the effect of the inhibitor was more pronounced, rescuing the plaque size of the mutant to that of the WT. Hence, the defect in intercellular spread of UL46null PRV can be rescued by inhibition of gap junction communication or by inhibition of STING, suggesting that pUL46-induced suppression of GJIC may benefit intercellular virus spread by suppressing activation of the antiviral STING pathway.

## Discussion

In the current study, we show that the most abundant connexin isoform, Cx43, becomes phosphorylated in PRV-infected cells, which correlates with suppression of GJIC. Future research may shed light on the phospho-sites targeted by PRV, as well as the mechanisms and functional consequences of Cx43 degradation at late stages of PRV infection. Mechanistically, infection with PRV results in ERK1/2 activation, caused by the viral pUL46 protein, which is essential for Cx43 phosphorylation and GJIC inhibition. This pUL46-induced suppression of GJIC is associated with enhanced viral cell-to-cell spread *in vitro*, possibly by limiting the transmission of antiviral messengers that trigger STING activation.

In addition to identifying viral and host proteins required for manipulation of host GJIC by an alphaherpesvirus, our data for the first time link virus-induced GJIC inhibition to enhanced virus intercellular spread. Indeed, pharmacological inhibition of GJIC as a way to mimic the inhibitory effect of pUL46 on GJIC rescued the reduced intercellular spread of UL46null PRV, while it did not affect the intercellular spread of WT PRV (Fig 5A and 5B, and S5A and S5B Fig). Pharmacological inhibition of STING also resulted in UL46null PRV plaque sizes that matched those formed by WT PRV (Fig 5C and 5D, and S5C and S5D Fig). We therefore speculate that PRV suppresses GJIC in order to limit the spread of messengers (such as cGAMP [9]) that would trigger activation of the antiviral STING pathway in neighbouring cells.

Viral interference with GJIC has been reported before for mammalian viruses, but the mechanisms underlying this inhibition and the consequences of GJIC inhibition remained largely unknown. For example, infection of astrocytes or meningeal fibroblasts with mouse hepatitis virus (MHV) leads to reduced expression of Cx43 and suppression of GJIC [44,45]. Zika virus (ZIKV) infection of cardiomyocytes triggers downregulation of Cx43, which is thought to reduce GJIC and contribute to heart disease in infected mice [46]. Classical swine fever virus (CSFV) downregulates GJIC in infected endothelium, accompanied by Cx43 phosphorylation and ERK1/2 activation [47]. Adenovirus type 5 infection triggers rapid Cx43 phosphorylation via an unknown mechanism [48]. As mentioned higher, also for the herpesviruses HCMV and HSV-2, earlier studies pointed to inhibition of GJIC, although the underlying mechanism and functional consequences remained unclear [14-17].

Interestingly, several of the viruses that suppress GJIC have been reported to activate ERK1/2 early in infection, and this ERK1/2 activation is often associated with enhanced viral spread and replication (CMV [49-52], ZIKV [53], CSFV [47,54], MHV [55,56], Ad5 [57,58]). Although speculative, this may point to potentially conserved mechanisms of virus-induced suppression of GJIC. Notably, ERK1/2-mediated Cx43 phosphorylation and consequent GJIC inhibition constitutes a key physiological regulation process resulting in GJ closure and internalization [31,59-64], indicating that viruses like PRV usurp this cellular process of GJIC regulation for their own benefit. It will be interesting in future research to identify the sites in Cx43 that are phosphorylated via PRV pUL46, and whether these sites are conserved in other viruses.

The PRV-induced ERK1/2 activation and Cx43 phosphorylation that we describe in infected ST cells and WB-F344 cells depends critically on the viral pUL46 protein. Earlier, we reported that at late stages of PRV infection in porcine PK-15 cells and T lymphocytes, the viral gE envelope protein of PRV also triggers ERK1/2 activation [32,33]. It will therefore be interesting in future assays to assess whether, in particular cell types and/or at particular time points in infection, gE may also affect Cx43 phosphorylation and GJ permeability or, alternatively, whether there is something specific about pUL46-induced ERK1/2 activation that leads to Cx43 phosphorylation. Thus far, most information on the functions of the alphaherpesvirus pUL46 tegument protein, also referred to as the VP11/12 protein, has been derived from studies on HSV-1. Of note, HSV-1 pUL46 has been reported to suppress STING-mediated IFN type I production by binding to both STING and TNK1 and by impairing the interaction between TBK1 and IRF3 [65,66]. PRV pUL46 has also been reported to interact with STING [67]. Further, HSV-1 pUL46 has been reported to activate the Akt signalling pathway in T lymphocytes and fibroblasts [68-71] and to trigger phosphorylation and degradation of Dok-2 in T cells [72]. In addition, in HSV-1-infected fibroblasts, pUL46 levels seem to be tightly regulated via the viral E3 ubiquitin ligase ICP0 [73].

Although our findings primarily reveal novel fundamental insights in virus-host interactions, one may speculate on the potential longer-term applications of these insights. On the one hand, these data may contribute to the rational design of attenuated virus vaccines that display reduced intercellular spread and possibly increased innate [9] and adaptive [12] immune responses. This would fit in similar approaches where vaccine candidates have been designed with the aim of minimizing the immune evasion capabilities of the pathogen [74-78]. On the other hand, the current findings may possibly have implications for advancements in oncolytic virotherapy. The single FDA/EMA-approved oncolytic viral vector thus far is talimogene laherparepvec (T-VEC), an HSV-1-based vector approved for treating unresectable metastatic melanoma [79]. Some HSV-based oncolytic virus vector candidates capitalize on the fact that, upon infection of tumor cells, the HSV-1 thymidine kinase (tk) is introduced into these malignant cells, rendering them susceptible to the cytotoxic effects of the prodrug ganciclovir (GCV). Intercellular GJ-mediated transfer of activated GCV from infected tumor cells to adjacent cells amplifies the effect of this treatment by inducing cell death in bystander malignant cells that do not express the HSV tk [80-85]. It has been reported that incorporating Cx43 alongside tk in the viral vector significantly boosts killing of bystander tumor cells in instances where cells exhibit diminished levels of GJIC [81]. Therefore, the current results may aid in the design of oncolytic viral vectors that maintain functional GJIC in infected cells.

We found that early in infection of WB-F344 cells with PRV strains that lack the viral pUL46 protein, GJIC is substantially increased compared to that observed in mock-infected cells. We hypothesize that this may represent a strategy of the host cell to warn its neighbours of the ongoing infection and to induce an antiviral state. PRV negates this upregulation by inducing pUL46- and ERK1/2-mediated phosphorylation of Cx43 and suppression of GJIC. Such scenario would underscore the sophisticated strategies employed by these viruses to subvert host cell signalling for their own benefit.

Taken together, these findings reveal a novel aspect of (herpes)virus biology and open avenues for the development of targeted therapies and antiviral strategies that retain or lack GJIC.

## Methods

### Ethics statement

Isolation of primary porcine epithelial kidney (PPK) cells was performed as described before [86]. Animal care and euthanasia for this purpose were performed according to Federation of European Laboratory Associations (FELASA) guidelines and approved by the Ethical Committee of the Faculty of Veterinary Medicine of Ghent University.

### Cells and viruses

Swine testicle epithelial (ST) cells (ATCC CRL-1746; *Sus scrofa*, pig) were cultured in Modified Eagle’s Medium (MEM) supplemented with 10% inactivated fetal bovine serum (FBS), 100 U/ml penicillin, 0.1 mg/ml streptomycin, 50 μg/ml gentamicin, and 1 mM sodium pyruvate (all from Gibco, Thermo Fisher Scientific, Waltham, MA, USA).

Swine kidney epithelial (SK-6) cells (RRID: CVCL_D296; *Sus scrofa*, pig) were cultured in MEM supplemented with 10% inactivated FBS, 100 U/ml penicillin, 0.1 mg/ml streptomycin, 50 μg/ml gentamicin, and 1 mM sodium pyruvate (all from Gibco, Thermo Fisher Scientific).

Rat liver epithelial cells (WB-F344) (RRID:CVCL_9806, *Rattus norvegicus*, rat) were purchased from Cell Lines Service (CLS) GmbH (Eppelheim, Germany), and cultured in Dulbecco’s Modified Eagle’s Medium (DMEM) supplemented with 10% FBS, 100 U/ml penicillin, 0.1 mg/ml streptomycin, 50 μg/ml gentamicin, and 1 mM sodium pyruvate (all from Gibco, Thermo Fisher Scientific).

The primary porcine epithelial kidney (PPK) cells were isolated from 7-week-old piglets (*Sus scrofa,* pig) as described before [86]. In brief, following the removal of the renal capsule and the outer layer of the kidney cortex, the intermediate layer of the kidney cortex was isolated and fragmented into small pieces (approx. 1 mm^3^) and sequentially digested with trypsin 2.5 mg/ml resulting in a single cell suspension. The primary kidney cells collected from the several rounds of trypsinization were cultured in PPK medium consisting of MEM (with L-glutamine) supplemented with 10% FBS, 100U/ml penicillin, 0.1 mg/ml streptomycin, 50 µg/ml gentamycin, 1.25 μg/ml amphotericin B (all from Gibco, Thermo Fisher Scientific), and 0.05% (v/w) lactalbumin hydrolysate (BD Biosciences, San José, CA) for 4 days. PPK cells were then trypsinized and seeded at 1,5 million cells/well in 6-well-plates. As a control, PPK cells were stained against the epithelial cell marker pan-cytokeratin (Dako, Agilent, Santa Clara, CA, catalog number m3515), which always indicated purity of > 95%.

The PRV strains used were NIA-3 WT [87], NIA-3 ΔUS3 [88], NIA-3 ΔUL13 [89] (all kindly donated by the ID-DLO, the Netherlands); Kaplan WT [90] and Kaplan ΔUL46 [40]; Becker WT [91] (kindly provided by L. Enquist, Princeton University, USA), and Becker ΔUL46 (PRV-GS7976) produced for the current studies. Briefly, the pBecker3 infectious clone [92] harbored in the GS1783 bacteria [93] was modified by two-step recombination [94]. Each mutation was produced using primers designed to amplify a kanamycin-resistance cassette flanked by I-SceI restriction sites that was inserted at the target coding sequence by RED-GAM homologous recombination targeted by the primer 5’ ends. The insertion was designed to partially duplicate the surrounding sequence, with the desired coding change. The resulting merodiploid intermediate was resolved by inducing cleavage of the I-SceI sites followed by a second round of RED-GAM homologous repair across the redundant flanking sequences. The UL46 deletion spanned codons 2-674 and inserted a stop codon in frame with the ATG (codon 1) at the deletion site. This design left the last 18 codons on UL46 intact but effectively non-coding because they were behind the inserted stop codon. Additional strains used were Kaplan WT [90] and Kaplan ΔUL46 [40].

Virus stocks were grown and titrated by serial dilution on ST cells. Every infection was performed on confluent cell monolayers and always using a multiplicity of infection of 10 plaque-forming units per cell (MOI of 10 PFU/cell), with the exception of the plaque assay, for which only 5,000 PFUs/well were used and the assay shown in Fig. 2F, where cells were infected with either an MOI of 10 or 1 PFU/cell.

### Inhibitors and reagents

The MEK1/2 inhibitor U0126 (catalog number tlrl-u0126) was purchased from InvivoGen (Toulouse, France). The gap junction inhibitor meclofenamate sodium (catalog number HY-B1320) was purchased from MedChemExpress (MCE) (Monmouth Junction, NJ). Lambda protein phosphatase (λ-PPse) (catalog number P0753S) was purchased from New England Biolabs (Ipswich, MA). The PI3 kinase inhibitor wortmannin (catalog number BML-ST415-0001) was purchased from Enzo Life Sciences (Farmingdale, NY). The 26S proteasome inhibitor MG-132 (catalogue number C2211) was purchased from Merck (Rahway, NJ). The autophagy inhibitor chloroquine diphosphate (catalog number 4109) was purchased from Tocris Bioscience (Bristol, United Kingdom). The E1 ubiquitin ligase inhibitor PYR41 (catalog number N2915) was purchased from Merck. The STING inhibitor H-151 (catalog number inh-h151) was purchased from InvivoGen. The gap junction-permeable fluorescent dye lucifer yellow CH (LY) (catalog number 25573) was purchased from Cayman Chemical (Ann Arbor, MI).

### Antibodies for the detection of PRV proteins

The antibodies used for the detection of PRV proteins were mouse anti-gE [95] (1:100 for WB), mouse anti-pUS3 [96] (1:100 for WB) (kindly provided by L. Enquist), rabbit anti-IE180 [97] (1:1,000 for WB)(kindly provided by E. Tabarés), mouse anti-pUL13 [98] (1:1,000 for WB)(kindly provided by L. Enquist), rabbit anti-pUL46 [40] (1:100,000 for WB), and mouse anti-gB [95] (1:50 for IF).

### Antibodies for the detection of cellular proteins

Horseradish peroxidase (HRP)-conjugated mouse anti-α-tubulin (1:2000 for WB, Abcam, Cambridge, United Kingdom, catalog number ab40742), rabbit anti-Cx43 (1:2,000 for WB, Abcam, catalog number ab11370), rabbit anti-total ERK1/2 (p44/42 MAPK) (1:1,000 for WB, Cell Signalling Technology, Danvers, MA, catalog number #9102), rabbit anti-phospho ERK1/2 (phospho-p44/42 MAPK) (Thr202/Tyr204) (1:1,000 for WB, Cell Signalling Technology, catalog number #9101).

### Western blotting assays

Samples were harvested in ice-cold 1x RIPA buffer, made from 10x RIPA buffer (catalog number ab156034, Abcam) diluted in ultrapure water containing protease inhibitor cocktail (cOmplete mini EDTA free, catalog number 11836170001, Roche, Basel, Switzerland) and phosphatase inhibitor cocktail (PhosStop, catalog number 4906845001, Roche). Cell lysates were kept at 4°C for 20 min before storage at -20°C. SDS-PAGE and Western blotting procedures were extensively described in [99]. The blocking solution was composed of 5% (w/v) non-fat dry milk diluted in 0.1% PBS-Tween 20 (PBS-T). However, for the detection of phosphorylated proteins, the blocking buffer used consisted of 5% (w/v) bovine serum albumin (BSA) (fraction V, catalog number 1120180100, Sigma-Aldrich, St. Louis, MI) diluted in PBS-T. Blotted PVDF membranes were blocked for 1h at room temperature (RT). Primary antibodies diluted in the corresponding blocking buffer were incubated with gentle shaking overnight at 4°C. Prior to the incubation with the secondary antibodies, three washing steps with PBS-T of 10 min each at RT were carried out. HRP-linked secondary antibodies, goat anti-IgG mouse-HRP (1:2,000, catalog number P0447, Dako) or goat anti-IgG rabbit-HRP (1:3,000, catalog number P0448, Dako) were diluted in the corresponding blocking solution and incubated at RT for 1h. After three washing steps, protein bands were visualized by chemiluminescence using a ChemiDoc imaging device (Bio-Rad, Hercules, CA). Depending on the levels of the targeted protein and the antibody sensitivity, ECL Plus substrate (GE Health Care, Chicago, IL) or SuperSignal West Femto maximum sensitivity substrate (Thermo Fisher Scientific) was used.

For the assessment of PRV-induced Cx43 phosphorylation in WB-F344 cells, PhosTag gels were used for Western Blotting (Fujifilm, Tokyo, Japan). PhosTag gels were employed as before in [100].

### Dephosphorylation assay

Samples were harvested in ice-cold dephosphorylation assay lysis buffer containing 10% Nonidet P-40 Substitute (Merck, catalog number 11332473001) and protease inhibitor cocktail (cOmplete mini EDTA free, catalog number 11836170001, Roche). Protein concentration was determined using the Bio-Rad protein assay kit (catalog number 5000001, Bio-Rad). 100 µg of protein per sample were used for the dephosphorylation assay, which was carried out according to the manufacturer’s instructions. Following dephosphorylation, samples were digested by adding 5x Laemmli Sample Buffer containing β-mercaptoethanol (catalog number 161-0737, Bio-Rad), and then heated at 94ºC for 5 minutes. Subsequently, Western blotting was performed as described above to assess protein mobility shifts induced by the dephosphorylation assay.

### Quantification of Cx43 phosphorylation

Using the Image Lab software (Bio-Rad), a box was drawn encompassing Cx43 in mock-infected cells. This box served as a guide for the base position of unphosphorylated Cx43. Then, an equal size box was drawn on top of unphosphorylated Cx43 and its intensity measured. This acted as the phosphorylated Cx43 region. To determine changes phosphorylation status in any given sample, the mean value of the phosphorylated region was divided between the value of the unphosphorylated region.

### Immunofluorescence assays

For gB visualization, cell monolayers were rinsed with sterile PBS (+ Ca, Mg) prior to fixation using 4% paraformaldehyde for 10 min at RT. Cells were then permeabilized using 0.1% Triton-X100 in PBS (+ Ca, Mg) for 10 minutes at RT. Then the samples were rinsed 3 times with PBS (+ Ca, Mg). Primary mouse anti-gB antibody was diluted 1:50 in PBS (+Ca, Mg) and incubated overnight at 4ºC. Following primary antibody incubation, the cells were rinsed 3 times with PBS (+Ca, Mg). The secondary antibody was goat anti-mouse IgG2a (Alexa Fluor 488) (Thermo Fisher Scientific, catalog number A-21131), which was diluted 1:200 in PBS (+Ca, Mg) and incubated for 1 hour at 37ºC. Following secondary antibody incubation, cells were rinsed 3 times with PBS (+Ca, Mg). The nuclei were counterstained with Hoechst 33342 (Thermo Fisher Scientific, catalog number H3570) diluted 1:200 in PBS (+ Ca, Mg) for 2 minutes at RT. Then, the cells were rinsed 3 more times with PBS (+ Ca, Mg) and imaged directly on MW6 plates.

All pictures shown were taken using a Thunder imaging system (Leica). The resulting images were analyzed using ImageJ imaging software (NIH, USA).

### Scrape loading-dye transfer (SL-DT) assay

The SL-DT assay was performed as described by Sovadinová and colleagues in [18]. Briefly, WB-F344 cells were rinsed twice with warm PBS (+ Ca, Mg). Then, 1 ml of Lucifer Yellow (LY) CH 1 mg/ml dissolved in sterile PBS (+ Ca, Mg) was applied to a well from a 6-well-plate. Then, a wound was performed on the monolayer of cells by gently scraping a surgical scalpel over the cells. The transient plasma membrane disruptions allow the cell-impermeable LY dye to be incorporated into the cells. Next, the cells were incubated for 3 minutes at RT and protected from light, allowing the cell-impermeable LY dye to spread to neighbouring cells via GJs. After this incubation step, LY was removed, the cells were washed 3 times with warm PBS (+ Ca, Mg), and then fixed using 4% PFA for 10 minutes at RT. The cells were then immediately imaged using the Leica Thunder system. Dye spread was measured in at least 4 scrapes/condition/independent repeat, using a custom ImageJ macro adapted from [101].

### Plaque assay

WB-F344 cells were cultured in 6-well-plates and infected with PRV at 5000 PFU/well. At 2 hpi, medium was replaced by 3 ml of semisolid medium consisting of WB-F344 medium supplemented with 2% methylcellulose (CMC). At 24 hpi, the CMC-containing medium was discarded, the cells were rinsed 3 times with PBS (+Ca, Mg), and then fixed using 4% PFA for 10 minutes at RT. For PRV plaque detection, the cells were immunostained against PRV gB following the procedure described above. At least 10 plaques per condition were measured during each experimental repeat.

### Statistical analysis

Statistical analyses were performed using Prism 6 (GraphPad Software). Statistical differences among the experimental groups were determined by unpaired t-tests.

## Supporting information

S1 Fig Gap junction-mediated fluorescent dye spread in WB-F344 cells is blocked by the GJIC inhibitor meclofenamate sodium. The scrape loading dye transfer (SL-DT) assay was performed in WB-F344 cells that were either (lower row) or not (upper row) pre-treated for 1h with 50 μM of the GJIC inhibitor meclofenamate sodium. In the inhibitor-treated sample, the Lucifer Yellow CH (LY) solution used for the SL-DT assay was also supplemented with 50 μM meclofenamate sodium. Scale bar: 100 μm.
(TIFF)

S2 Fig PRV-induced Cx43 phosphorylation is conserved across diverse infectious strains and different porcine epithelial cells. (A) ST cells were infected with WT PRV strain NIA-3 (MOI, 10 PFU/cell). At 8 hpi, the cells were treated or not with 10 μM of the UAE1 ubiquitin ligase inhibitor PYR41. Cell lysates were harvested at 17 hpi and subjected to Western blot analysis. (B) Western blot analysis of Cx43 phosphorylation in ST cells infected with WT PRV strains NIA-3, Becker, or Kaplan (MOI, 10 PFU/cell) at 4 hpi. (C) Western blot analysis of Cx43 phosphorylation during a time-course assay in SK-6 cells infected with WT PRV strain NIA-3 (MOI, 10 PFU/cell) from 0 to 24 hpi. (D) Western blot analysis of Cx43 phosphorylation during a time-course assay in PPK cells infected with WT PRV strain NIA-3 (MOI, 10 PFU/cell) from 0 to 12 hpi. All Western blots shown in this figure are representative examples from three independent repeats of each experiment.
(TIFF)

S3 Fig UL46null PRV replication efficiency is comparable to that of WT PRV. ST cell monolayers were infected at a multiplicity of infection of 10 PFU/cell with WT or UL46null PRV, either in Becker or Kaplan genetic background. At 2 hpi, the cells were treated with sodium citrate buffer (pH 3.0; 40 mM sodium citrate, 10 mM KCl, 135 mM NaCl) for 2 min at room temperature to inactivate remaining infectious virus from the inoculum. At 16 and 24hpi, the supernatants were harvested. The supernatants containing infectious progenies were titrated by 1/10 serial dilution assays on ST cells seeded on 96-well plates, performed in quadruplicate. PRV-induced cytopathic effect served as a readout. Extracellular virus titers are expressed as the number of PFU/ml on a logarithmic scale. Graphs represent mean and standard deviations of three independent repeats (‘ns’ not significant).
(TIFF)

S4 Fig pUL46-dependent ERK1/2 activation, Cx43 phosphorylation and suppression of GJIC in PRV-infected cells is cross-strain reproduceable and pUL46-dependent ERK1/2 activation and Cx43 phosphorylation also occurs in WB-F344 cells. (A) Western blot analysis of Cx43 phosphorylation and ERK1/2 activation in ST cells infected with WT PRV strain Kaplan or an isogenic UL46null strain at 4 and 8 hpi (MOI, 10 PFU/cell). (B) Western blot analysis of Cx43 phosphorylation and ERK1/2 activation during a time-course assay in WB-F344 cells infected with WT PRV strain Becker or an isogenic UL46null strain (MOI, 10 PFU/cell) from 0 to 16 hpi. (C) SL-DT assay performed in WB-F344 cells that were mock-infected or infected for 8 h with WT PRV strain Kaplan or with an isogenic UL46null strain (MOI, 10 PFU/cell). Scale bar: 100 μm. (D) Quantitative analysis of the SL-DT assay shown in S4C Fig. (E) SL-DT assay performed in WB-F344 cells that were mock-infected or infected for 16 h with WT PRV strain Kaplan or with an isogenic UL46null strain (MOI, 10 PFU/cell). Scale bar: 100 μm (F) Quantitative analysis of the SL-DT assay shown in S4E Fig. For the quantification of SL-DT assays dye spread area was normalized to dye spread in mock-infected cells (set to 1). Graphs represent mean and standard deviations of three independent repeats (‘ns’ not signifficant, ‘****’ P ≤ 0.0001). All Western blot and fluorescence images shown in this figure were taken from one representative assay out of three independent repeats of each experiment.
(TIFF)

S5 Fig The impaired cell-to-cell spread of UL46null PRV, and its rescue by GJIC or STING inhibition, is cross-strain reproduceable. (A) Plaque assay performed in WB-F344 cells infected with PRV WT strain Kaplan or an isogenic UL46null mutant. 5,000 plaque forming units (PFUs) were used per well of a 6-well-plate to infect WB-F344 cells. At 2 hours post-inoculation, WB-F344 medium containing 2% carboxymethyl cellulose and supplemented or not with 50 μM of the GJIC inhibitor meclofenamate sodium was added. Cells were fixed at 24 hpi, and stained against PRV gB. Scale bar: 50 μm. (B) Quantitative analysis of plaque sizes shown in S5A Fig (Y-axis represents number of pixels per plaque (ppp)). (C) Plaque assay performed in WB-F344 cells infected with PRV WT strain Kaplan or an isogenic UL46null mutant. 5,000 plaque forming units (PFUs) were used per well of a 6-well-plate to infect WB-F344 cells. At 2 hours post-inoculation, WB-F344 medium containing 2% carboxymethyl cellulose and supplemented or not with 15 μM of the STING inhibitor H-151 was added. Cells were fixed at 24 hpi, and stained against PRV gB. Scale bar: 50 μm. (D) Quantitative analysis of plaque sizes shown in S5C Fig (Y-axis represents number of pixels per plaque (ppp)). Graphs represent mean and standard deviations of three independent repeats (‘ns’ not significant, ‘**’ P ≤ 0.01, ‘***’ P ≤ 0.001 ‘****’ P ≤ 0.0001). Immunofluorescence images shown in this figure were taken from one representative assay out of three independent repeats of each experiment. At least 10 plaques per condition were measured during each experimental repeat.
(TIFF)

S1 Data Data values behind the means and standard deviations that were used to build the different graphs. Different worksheet tabs contain data of SL-DT assays, Cx43 phosphorylation quantifications, virus titers, and plaque sizes and are ordered according to the order in which the corresponding (supplementary) figures are cited in the manuscript text.
(XLSX)
