# Supplementary figures and images for: Pseudorabies virus infection triggers pUL46-mediated phosphorylation of connexin-43 and closure of gap junctions to promote intercellular virus spread
Source: PLoS Pathog. 2025 Jan 21;21(1):e1012895. doi: 10.1371/journal.ppat.1012895 (PMC11774492; doi:10.1371/journal.ppat.1012895)

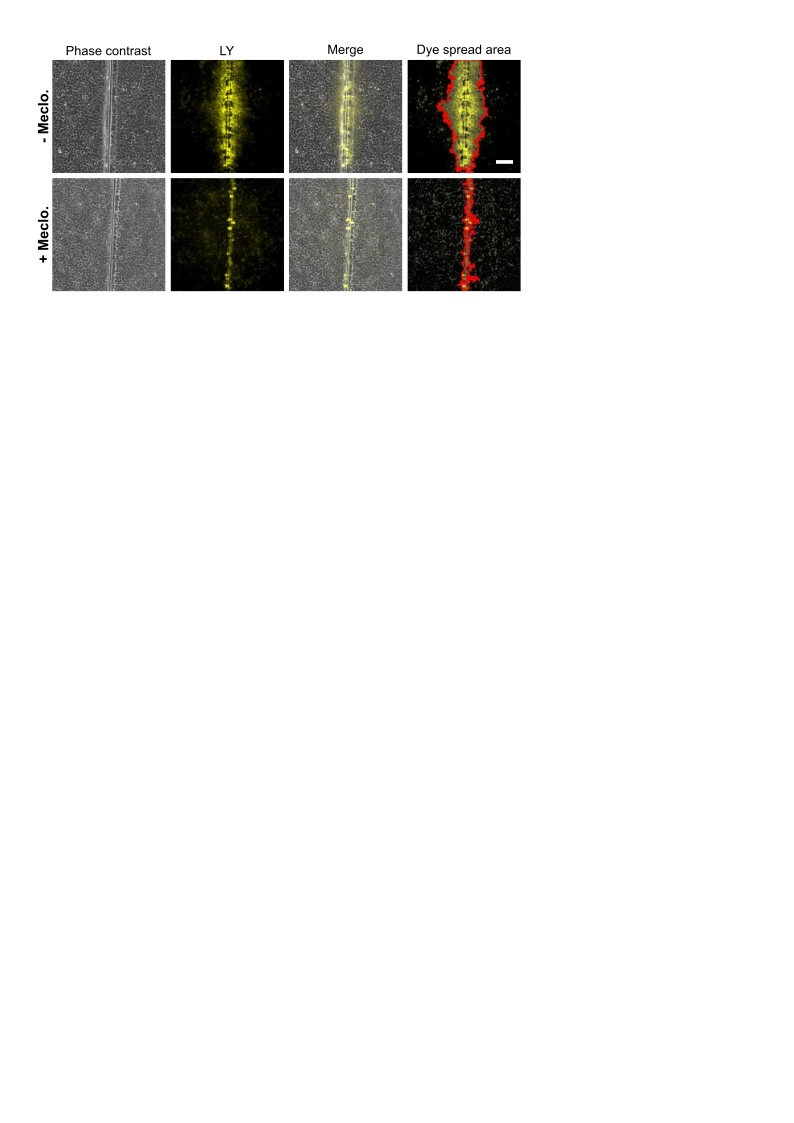

Supplement: S1 Fig — The scrape loading dye transfer (SL-DT) assay was performed in WB-F344 cells that were either (lower row) or not (upper row) pre-treated for 1h with 50 μM of the GJIC inhibitor meclofenamate sodium. In the inhibitor-treated sample, the Lucifer Yellow CH (LY) solution used for the SL-DT assay was also supplemented with 50 μM meclofenamate sodium. Scale bar: 100 μm. (TIFF) [file ppat.1012895.s001.tiff]

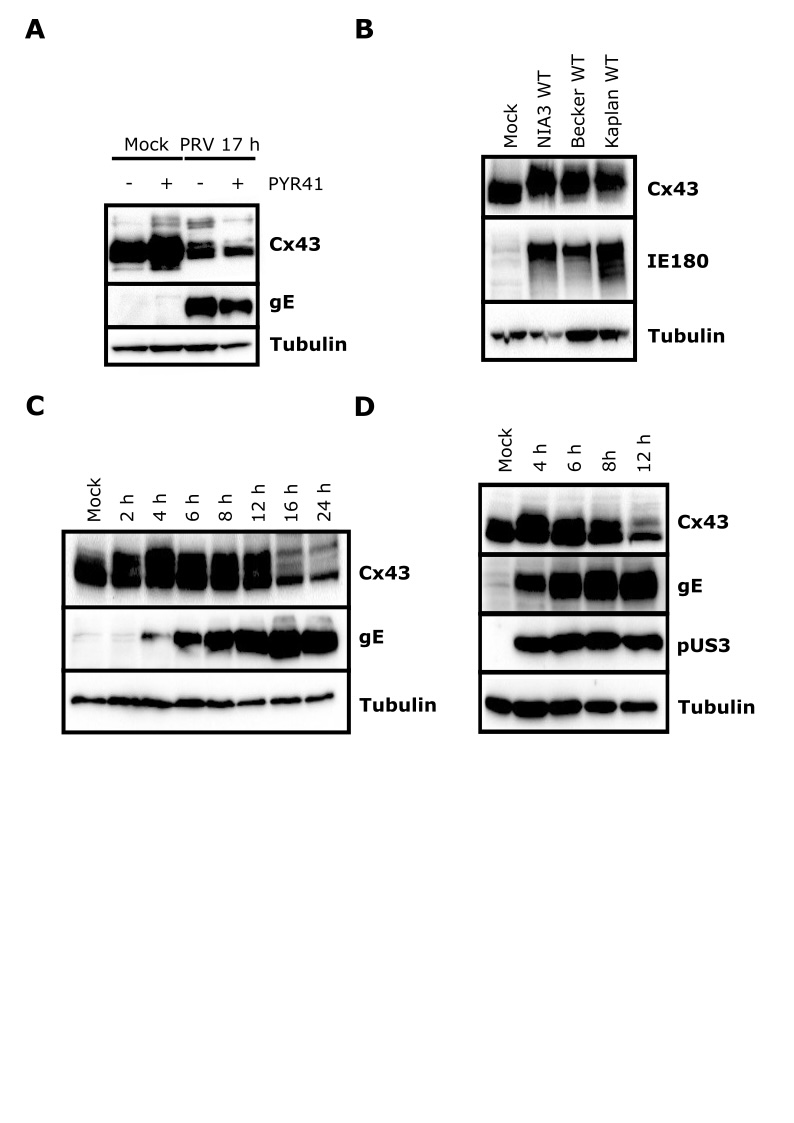

Supplement: S2 Fig — (A) ST cells were infected with WT PRV strain NIA-3 (MOI, 10 PFU/cell). At 8 hpi, the cells were treated or not with 10 μM of the UAE1 ubiquitin ligase inhibitor PYR41. Cell lysates were harvested at 17 hpi and subjected to Western blot analysis. (B) Western blot analysis of Cx43 phosphorylation in ST cells infected with WT PRV strains NIA-3, Becker, or Kaplan (MOI, 10 PFU/cell) at 4 hpi. (C) Western blot analysis of Cx43 phosphorylation during a time-course assay in SK-6 cells infected with WT PRV strain NIA-3 (MOI, 10 PFU/cell) from 0 to 24 hpi. (D) Western blot analysis of Cx43 phosphorylation during a time-course assay in PPK cells infected with WT PRV strain NIA-3 (MOI, 10 PFU/cell) from 0 to 12 hpi. All Western blots shown in this figure are representative examples from three independent repeats of each experiment. (TIFF) [file ppat.1012895.s002.tiff]

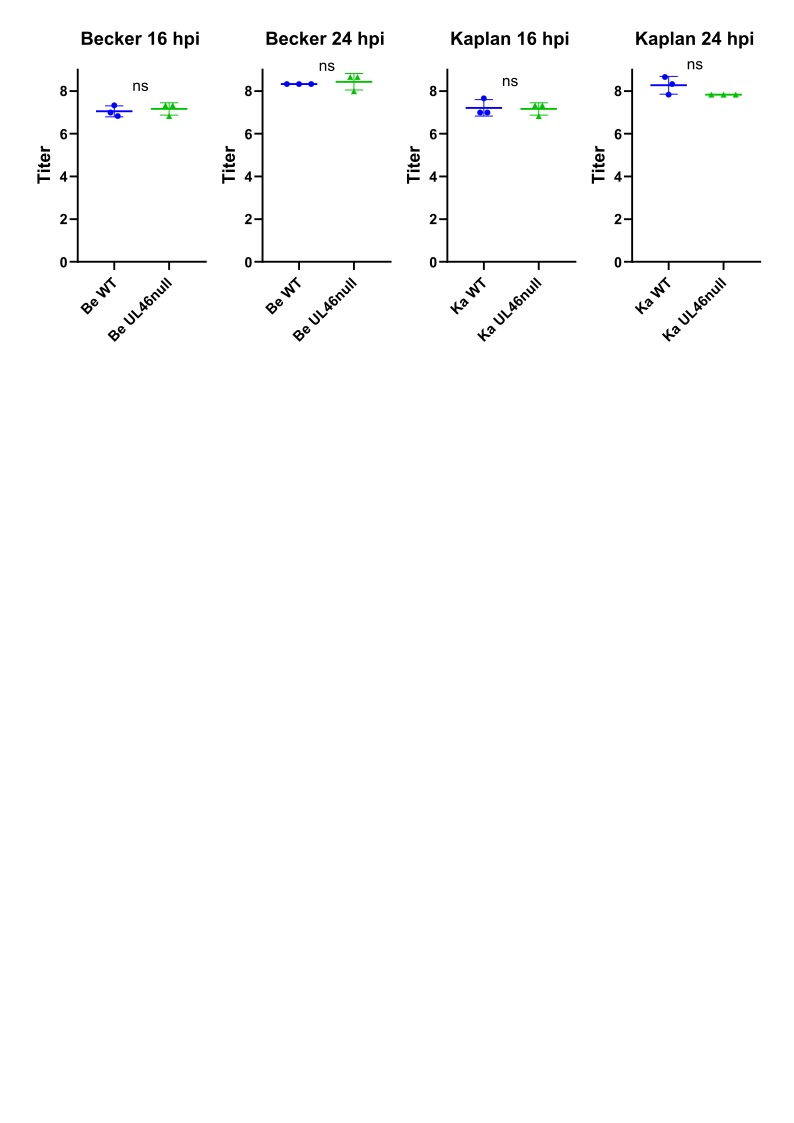

Supplement: S3 Fig — ST cell monolayers were infected at a multiplicity of infection of 10 PFU/cell with WT or UL46null PRV, either in Becker or Kaplan genetic background. At 2 hpi, the cells were treated with sodium citrate buffer (pH 3.0; 40 mM sodium citrate, 10 mM KCl, 135 mM NaCl) for 2 min at room temperature to inactivate remaining infectious virus from the inoculum. At 16 and 24hpi, the supernatants were harvested. The supernatants containing infectious progenies were titrated by 1/10 serial dilution assays on ST cells seeded on 96-well plates, performed in quadruplicate. PRV-induced cytopathic effect served as a readout. Extracellular virus titers are expressed as the number of PFU/ml on a logarithmic scale. Graphs represent mean and standard deviations of three independent repeats (‘ns’ not significant). (TIFF) [file ppat.1012895.s003.tiff]

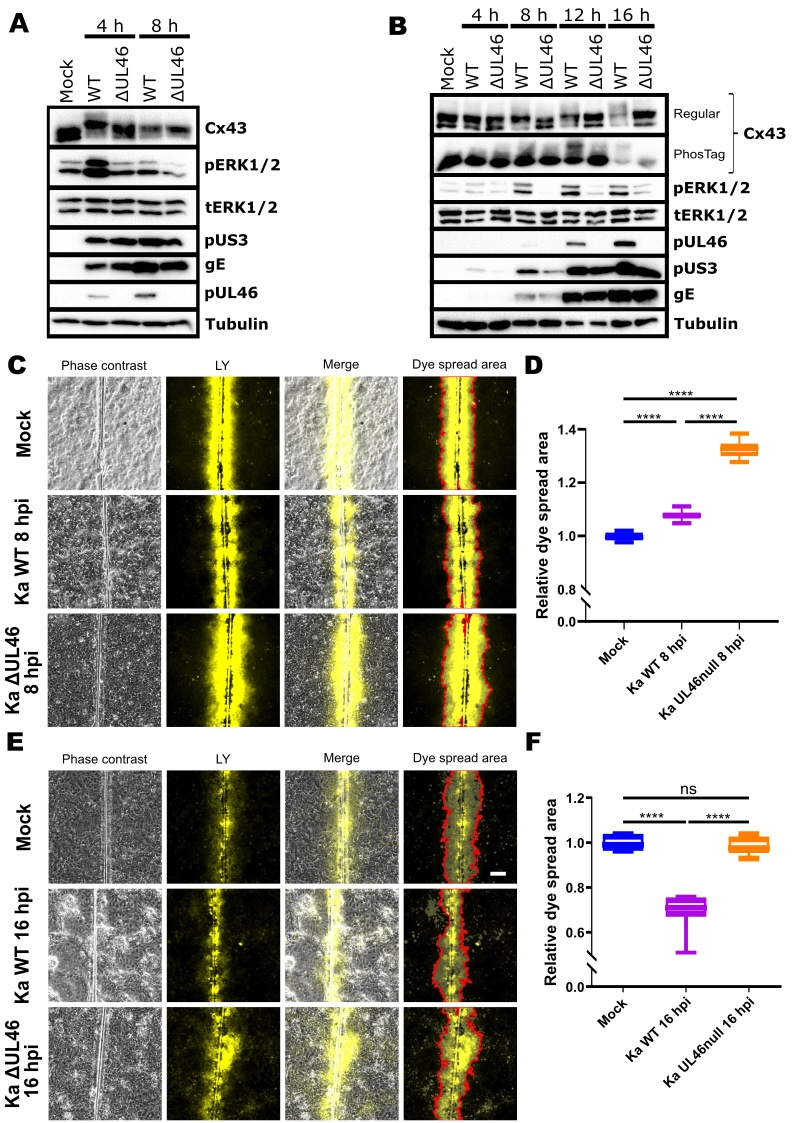

Supplement: S4 Fig — (A) Western blot analysis of Cx43 phosphorylation and ERK1/2 activation in ST cells infected with WT PRV strain Kaplan or an isogenic UL46null strain at 4 and 8 hpi (MOI, 10 PFU/cell). (B) Western blot analysis of Cx43 phosphorylation and ERK1/2 activation during a time-course assay in WB-F344 cells infected with WT PRV strain Becker or an isogenic UL46null strain (MOI, 10 PFU/cell) from 0 to 16 hpi. (C) SL-DT assay performed in WB-F344 cells that were mock-infected or infected for 8 h with WT PRV strain Kaplan or with an isogenic UL46null strain (MOI, 10 PFU/cell). Scale bar: 100 μm. (D) Quantitative analysis of the SL-DT assay shown in S4C Fig. (E) SL-DT assay performed in WB-F344 cells that were mock-infected or infected for 16 h with WT PRV strain Kaplan or with an isogenic UL46null strain (MOI, 10 PFU/cell). Scale bar: 100 μm (F) Quantitative analysis of the SL-DT assay shown in S4E Fig. For the quantification of SL-DT assays dye spread area was normalized to dye spread in mock-infected cells (set to 1). Graphs represent mean and standard deviations of three independent repeats (‘ns’ not signifficant, ‘****’ P ≤ 0.0001). All Western blot and fluorescence images shown in this figure were taken from one representative assay out of three independent repeats of each experiment. (TIFF) [file ppat.1012895.s004.tiff]

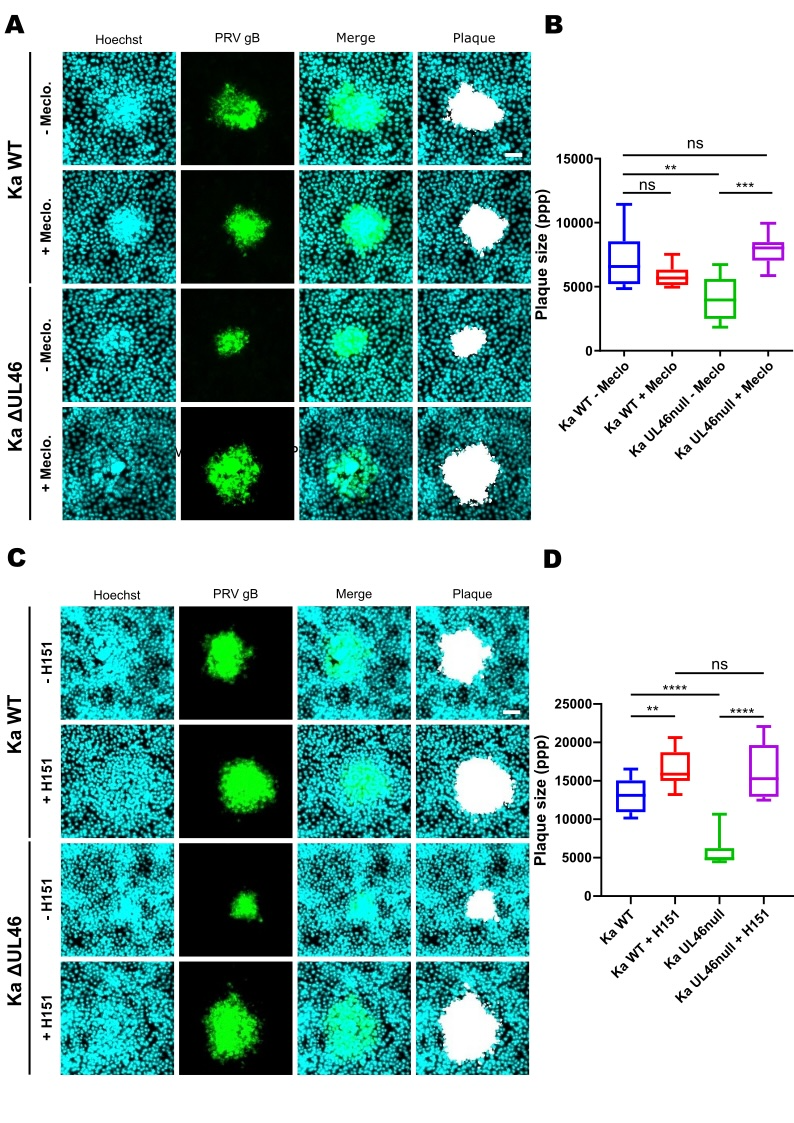

Supplement: S5 Fig — (A) Plaque assay performed in WB-F344 cells infected with PRV WT strain Kaplan or an isogenic UL46null mutant. 5,000 plaque forming units (PFUs) were used per well of a 6-well-plate to infect WB-F344 cells. At 2 hours post-inoculation, WB-F344 medium containing 2% carboxymethyl cellulose and supplemented or not with 50 μM of the GJIC inhibitor meclofenamate sodium was added. Cells were fixed at 24 hpi, and stained against PRV gB. Scale bar: 50 μm. (B) Quantitative analysis of plaque sizes shown in S5A Fig (Y-axis represents number of pixels per plaque (ppp)). (C) Plaque assay performed in WB-F344 cells infected with PRV WT strain Kaplan or an isogenic UL46null mutant. 5,000 plaque forming units (PFUs) were used per well of a 6-well-plate to infect WB-F344 cells. At 2 hours post-inoculation, WB-F344 medium containing 2% carboxymethyl cellulose and supplemented or not with 15 μM of the STING inhibitor H-151 was added. Cells were fixed at 24 hpi, and stained against PRV gB. Scale bar: 50 μm. (D) Quantitative analysis of plaque sizes shown in S5C Fig (Y-axis represents number of pixels per plaque (ppp)). Graphs represent mean and standard deviations of three independent repeats (‘ns’ not significant, ‘**’ P ≤ 0.01, ‘***’ P ≤ 0.001 ‘****’ P ≤ 0.0001). Immunofluorescence images shown in this figure were taken from one representative assay out of three independent repeats of each experiment. At least 10 plaques per condition were measured during each experimental repeat. (TIFF) [file ppat.1012895.s005.tiff]
